# Primary Malignant Melanoma of the Gallbladder: A Case Report and Review of the Literature

**DOI:** 10.1155/2012/693547

**Published:** 2012-10-11

**Authors:** Mehmet Fatih Haskaraca, Mustafa Ozsoy, İsmail Özsan, Kamile Kurt

**Affiliations:** ^1^Department of General Surgery, Merkez Efendi State Hospital, 35630 Manisa, Turkey; ^2^Department of General Surgery, Izmir University, Izmir, Turkey; ^3^Department of Pathology, Merkez Efendi State Hospital, Manisa, Turkey

## Abstract

Malignant melanoma is characterized by the ability of diffuse metastases. Since the first report of an isolated malignant melanoma case of the gallbladder, it is already controversial whether isolated cases are metastatic or primary tumors. A 49-year-old woman appealed to the emergency unit because of abdominal pain. Ultrasonography revealed increased thickness of the gallbladder wall and a lesion with surrounding fluid sized 12 mm without acoustic shadow, which arose from the gallbladder wall and was consistent with a polyp. Histopathologic evaluation of the surgical specimen after laparoscopic cholecystectomy revealed malign epithelial tumor consisting of atypical cells with large eosinophilic cytoplasm and dense melanin pigment within the cytoplasm of the tumor cells. As no other focus was identified as a result of the evaluation, the patient was diagnosed with primary malignant melanoma of the gallbladder. In this paper, we aimed to define our treatment modality for a case with isolated malignant melanoma of the gallbladder.

## 1. Background

Malignant melanoma is discussed among the malignancies with the least survival rates, which is known as its aggressive course and the capacity to make diffuse metastases. Metastasis to the gastrointestinal system occurs only in 2–4% of the patients with malignant melanoma [1]. In the gastrointestinal system, metastases most commonly occur in the intestines (35–65%), colon (5–9%), and in the stomach (5–7%). After Hambi and Wieting's report of the case with malignant melanoma arisen from the epithelium of the gallbladder in 1907, the interest for the malignant melanoma of the gastrointestinal system has been increased [2]. It is already controversial if the gallbladder involvement of the malignant melanoma is primary or secondary. The opinion accepted by most authors is that all malignant melanomas of the gallbladder are metastatic [3, 4]. However, there are occasional case reports of primary malignant melanoma arisen from the gallbladder in the literature. Another issue is the treatment protocol to implement. In the present article, we aimed to present a patient with primary malignant melanoma arisen from the gallbladder, which was diagnosed incidentally, and our treatment modality.

## 2. Case Presentation

**Figure 1:**
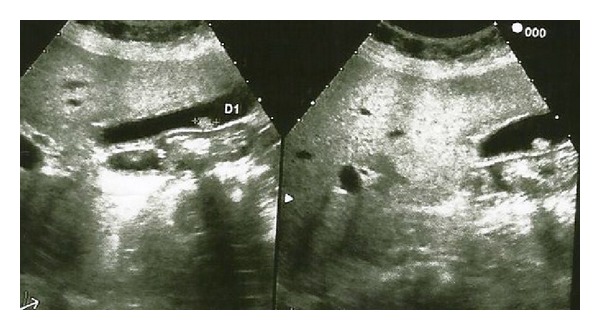
Image of polypoid mass attached to the gallbladder wall not showing acoustic shadow.

**Figure 2:**
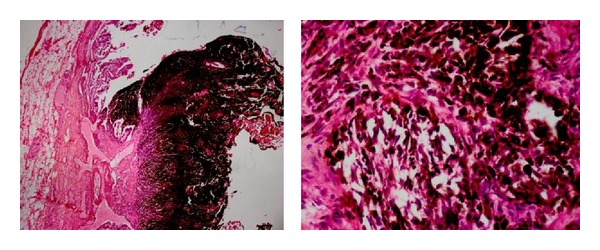
Malign epithelial tumor consisting of atypical cells with large eosinophilic cytoplasm, vesicular nucleus, and prominent nucleolus, which showed infiltration nesting beneath the epithelium of the gallbladder. A dense melanin pigment within the cytoplasm of the tumor cells was also noted.

A 49-year-old woman appealed to the emergency unit because of abdominal pain, nausea, and vomiting. There was not any peculiarity in her own and family medical history. On physical examination, peritoneal tenderness was found in the right upper abdominal quadrant. Laboratory tests revealed leukocytosis (16 000/mm^3^), other tests were as follows; serum aspartate aminotransferase, 15 IU/L (<30 IU/L) and alanine aminotransferase, 25 IU/L (<30 IU/L); serum alkaline phosphatase, 140 IU/L (100–320 IU/L); serum g-glutamyl transpeptidase, 20 IU/L (16–73 IU/L); and the serum bilirubin, 08 mg/dL (<1 g/dL). Abdominal ultrasonography revealed increased thickness of the gallbladder wall (8 mm) and a lesion with surrounding fluid sized approximately 12 mm without acoustic shadow, which arose from the gallbladder wall and was consistent with a polyp (Figure 1). The patient was diagnosed with acute cholecystitis depending on the clinical signs and laboratory tests. Her complaints resolved after medical treatment and the patient underwent elective laparoscopic cholecystectomy surgery. After intra-abdominal exploration, Calot's triangle was prepared. Cystic duct and artery were isolated, clipped, and cut. The gallbladder was mobilized through retrograde subserous removal and taken out of the abdomen with an endobag. The patient did not experience any surgical problem after laparoscopic cholecystectomy surgery and was discharged on the postoperative first day. Histopathologic examination of the surgical specimen revealed malign epithelial tumor consisting of atypical cells with large eosinophilic cytoplasm, vesicular nucleus, and prominent nucleolus, which showed infiltration nesting beneath the epithelium of the gallbladder. A dense melanin pigment accumulation within the cytoplasm of the tumor cells was noted (Figure 2). On the histopathologic examination of the gallbladder block, S100, Vimentin, and HMB-45 were found to be positive. The patient was diagnosed with malignant melanoma depending on these data. Further examinations were done to determine if malignant melanoma of the gallbladder was primary or metastatic (detailed physical examination, anamnesis, endosonographic procedures, ocular and dermatologic examinations, and positron emission tomography). The patient was diagnosed with primary malignant melanoma of the gallbladder, since there was no other focus on the evaluation. Chemoimmunotherapy was planned after surgery. The patient died on the 18th month of follow up because of the hemorrhage due to cranial metastasis.

## 3. Discussion

Tumor histobiology of malignant melanoma (MM) with prominent cutaneous origin still has obscurities. In MM, many patients have short survival because of diffuse metastatic disease and patient loss can be encountered years after the regression of the primary tumor because of the diffuse metastatic foci [5, 6]. MM is able to metastasize to all organs; it often metastasizes to the intestinal system. Among intestinal system metastases, malignant melanoma metastases to the gallbladder are particularly important [7, 8]. In the literature, there are MM cases primarily originated from the gallbladder along with the reports regarding the malignant melanoma metastases to the gallbladder. A case with malignant melanoma of the gallbladder was first reported by Jones in 1907 [2]. In the cases with primary or metastatic malignant melanoma of the gallbladder, which are very rare, the main concern is whether the lesion is primary or metastatic. Many authors argue that all malignant melanomas of the gallbladder are metastatic disease on the basis of embryologic development data and they denote that there is a main principal focus unidentified or regressed in the majority of the cases defined as primary [2, 9]. For malignant melanoma of the gallbladder, which cannot be identified histopathologically as primary or metastatic, diagnostic criteria for primary malignant melanoma of the gallbladder have been established by Allen and Spitz and revised by Heath and Womack [10]. A group of authors, primarily the authors mentioned above, advocated that the involvement of the gallbladder by malignant melanoma might be primary. They indicated the melanocytes as the source, which originate from the neural crest and migrate to the endoderm during the embryologic growth [11, 12]. According to the criteria, primary tumor must principally be solitary and arise from the mucosa. Primary tumor may also be papillary or polypoid. Besides, the most important diagnostic criterion is the junctional activity of the tumor on histopathologic examination (junctional activity defined as intraepithelial dissemination involves the presence of pigmented dendritic cells at the junction of the epithelium and lamina propria). As the final diagnostic criterion, primary malignant melanoma of the gallbladder is established when no other focus can be identified [13, 14]. Depending on a study conducted at the Memorial Sloan-Kettering Cancer Center, it can be said that malignant melanomas of the gallbladder are usually asymptomatic [15]. However, involvement of the main cystic duct may lead to obstructive jaundice or the symptoms mimicking acute cholecystitis due to the obstruction of the cystic duct. Also, the patients may present with right upper quadrant pain, weight loss, nausea, and vomiting [8, 16, 17]. In contrast to general belief, malignant melanomas of the gallbladder are not associated with gallstones and usually they are recognized incidentally during imaging scans [11]. Our patient presented with the clinical picture of acute cholecystitis unrelated to cholelithiasis, as seen in the literature. Among the diagnostic tools, ultrasonography is the cheapest and the most rapid method. Ultrasonography generally reveals sole or multiple infiltrative polypoid lesions not showing acoustic shadow, larger than 1 cm, and attached to the gallbladder wall [11]. Doppler ultrasonography also demonstrates remarkably increased blood flow. Besides ultrasonography, lesions are identified thoroughly on computed tomography scan, magnetic resonance imaging, and Positron Emission Tomography [18–20]. In MM cases, in which prognosis is poor, treatment options vary depending on the extensiveness of the disease and clinical picture of the patient. The outcome widely accepted by the authors is an average survival of 8.4 months for a malignant melanoma patient with metastatic disease [21]. With combined treatment modalities, survival prolongs up to 20 months for the cases accepted as primary. For the patients with localized malignant melanoma including primary malignant melanoma of the gallbladder, treatment modalities are still unclear because of insufficient number of patients. In a study with 19 patients, Dong et al. achieved more than 1 year survival after surgical treatment in 2 patients with isolated malignant melanoma of the gallbladder [11]. Patient series of the researchers named Velez, Seelig, and Kohler exemplify patient series with malignant melanoma of the gallbladder including metastatic cases. In these series, average survival time varies, but is less than 1 year [8, 22, 23]. In the cases with primary malignant melanoma of the gallbladder, another controversial issue is the surgical treatment procedure. It is questioned whether cholecystectomy should be performed using conventional or laparoscopic technique. Many authors prefer conventional cholecystectomy. They justify this choice with trocar recurrence seen in 2 of 3 patients after laparoscopic surgery performed by Katz et al. [15] However, in the majority of these patients, primary disease diagnosis is not known before surgery, and therefore the risk of iatrogenic intraoperative dissemination markedly increases in both open and laparoscopic surgeries. Another issue is the extent of the surgical dissection. Basically, the cases with malignant melanoma of the gallbladder with diffuse metastases should be considered as grade 4 and for these patients, palliative cholecystectomy rather than aggressive surgery should be performed to sustain patient comfort [21, 23, 24]. But for the patients with isolated malignant melanoma of the gallbladder, we think that surgical treatment option should be conventional cholecystectomy and lymph node dissection, that is, hepatoduodenal lymph node dissection, should be done with surgery as aggressive as possible. However, in our patient we did not have any idea about the diagnosis before the operation. Hence, we performed palliative cholecystectomy instead of aggressive dissection, as in many cases with malignant melanoma of the gallbladder. We should have performed an extended surgical dissection with the help of intraoperative frozen histopathologic examination. After this case, we started to take the gallbladder out the abdomen with endobag during laparoscopic cholecystectomy for polypoid lesions of the gallbladder. In this way, we can minimize the risk of trocar recurrence. Chemoimmunotherapy for malignant melanoma cases is also unclear. Chemoimmunotherapy is a palliative treatment procedure for grade 4 patients. It prolongs disease-free survival, but does not have any effect on 5-year survival rates and the rate of 5-year survival is approximately 5% [25]. There is a need for further studies about this issue. Current studies propose the use of high-dose interferon. However, there is no immunologic or chemotherapeutic agent available today, which is proven to ensure advantages on survival after resection in primary or metastatic melanoma [26].

## 4. Conclusion

 Whether isolated lesion is primary or metastatic in malignant melanoma cases with aggressive course and poor survival outcomes is a matter of debate. However, according to the common opinion, aggressive surgery seems to be a unique treatment modality contributing to survival rates in isolated malignant melanoma like malignant melanoma of the gallbladder. For all other cases, surgical treatment should aim to maintain life comfort.
